# Diagnosis of Parapneumonia Pleural Effusion with Serum and Pleural Fluid Cell-Free DNA

**DOI:** 10.1155/2019/5028512

**Published:** 2019-03-04

**Authors:** Chih-Min Su, Chia-Te Kung, Sheng-Yuan Hsiao, Nai-Wen Tsai, Yun-Ru Lai, Chih-Cheng Huang, Hung-Chen Wang, Wei-Che Lin, Ben-Chung Cheng, Yu-Jih Su, Cheng-Hsien Lu

**Affiliations:** ^1^Department of Emergency Medicine, Kaohsiung Chang Gung Memorial Hospital, Chang Gung University College of Medicine, Kaohsiung, Taiwan; ^2^Chung Shan Medical University, School of Medicine, Taiwan; ^3^Department of Biological Science, National Sun Yat-Sen University, Kaohsiung, Taiwan; ^4^Departments of Neurology, Kaohsiung Chang Gung Memorial Hospital, Chang Gung University College of Medicine, Kaohsiung, Taiwan; ^5^Neurosurgery, Kaohsiung Chang Gung Memorial Hospital, Chang Gung University College of Medicine, Kaohsiung, Taiwan; ^6^Radiology, Kaohsiung Chang Gung Memorial Hospital, Chang Gung University College of Medicine, Kaohsiung, Taiwan; ^7^Internal Medicine, Kaohsiung Chang Gung Memorial Hospital, Chang Gung University College of Medicine, Kaohsiung, Taiwan; ^8^Center for Shockwave Medicine and Tissue Engineering, Kaohsiung Chang Gung Memorial Hospital, Chang Gung University College of Medicine, Kaohsiung, Taiwan; ^9^Department of Neurology, Xiamen Chang Gung Memorial Hospital, Xiamen, Fujian, China

## Abstract

**Objective:**

As cell-free DNA levels in the pleural fluid and serum of parapneumonic pleural effusion (PPE) patients have not been thoroughly explored, we evaluated their diagnostic potential.

**Methods:**

Twenty-two PPE and 16 non-PPE patients were evaluated. Serum and pleural fluids were collected, and cell-free DNA was quantified. All biomarkers were assessed for correlation with days after admission. Receiver operating characteristic (ROC) curve analysis was used to determine diagnostic accuracy and optimal cut-off point.

**Results:**

Nuclear and mitochondrial DNA levels in the pleural fluid and nuclear DNA levels in serum of PPE patients were significantly higher than in those of the non-PPE patients. However, only cell-free DNA levels in pleural fluid correlated with days after admission among PPE patients (r= 0.464, 0.538, respectively). ROC curve analysis showed that nuclear and mitochondrial DNA in pleural fluid had AUCs of 0.945 and 0.889, respectively. With cut-off values of 134.9 and 17.8 ng/ml for nuclear and mitochondrial DNA in pleural fluid, respectively, 96% sensitivity and 81% specificity were observed for PPE diagnosis.

**Conclusion:**

Nuclear and mitochondrial DNA in pleural fluid possess PPE diagnostic potential and correlated with disease severity. Serum nuclear DNA could also be used to distinguish freshly admitted PPE patients (Day 1) from non-PPE patients, but with less accuracy.

## 1. Introduction

It is critical to determine the cause of pleural effusion because means of treatment is decided based on etiology of the disease [1]. Among known etiologies, correct diagnosis of parapneumonic pleural effusion (PPE) is most important, because in addition to appropriate antibiotics, patients sometimes need adequate drainage or surgical intervention to cure the disease [2]. Some studies have reported that up to 40% of bacterial pneumonia includes PPE [3]. Although small PPEs may be resolved solely by treatment with antibiotics, some patients will develop complicated PPE and/or empyema. Dean et al. showed that patients admitted to emergency rooms with pneumonia and pleural effusion had higher mortality rates and longer hospital stays [4].

Traditionally, diagnosis of PPE involves quantifying serum and pleural fluid lactate dehydrogenase (LDH) and total protein for Light's Criteria Rule to differentiate exudate and transudate [5]. Diagnosis of complicated PPE or empyema involves a white blood cell count, a gram stain of pleural fluid, and evaluation of pleural fluid pH. These biomarkers have different sensitivities and specificities for diagnosis of PPE, but all of them are deficient in some way [6].

Several new biomarkers have been investigated for diagnosis of PPE in recent years, including pleural fluid procalcitonin, C-reactive protein, and cell-free DNA [7–10]. Cell-free DNA resulting from programmed cell death, apoptosis, and rupture of blood cells or pathogens has proven useful in determining severity and prognosis for a variety of diseases [11, 12]. Cell-free DNA can exist in biological fluids other than plasma such as cerebrospinal and pleural fluids. Although pleural fluid cell-free DNA has had a higher area under curve (AUC) (0.904-0.950) for diagnosis of PPE in previous studies, case numbers have been low, and the relationship of pleural fluid cell-free DNA levels with PPE severity has not been adequately addressed [10, 13]. The aim of this study was to evaluate the use of both serum and pleural fluid cell-free DNA levels for diagnosis of PPE and to determine whether these levels correlate with severity of PPE.

## 2. Patients and Methods

### 2.1. Study Population

This was a prospective study of changes in cell-free DNA levels in blood and pleural fluid over time in patients with pleural effusion and an assessment of the correlation of the DNA levels with disease severity in parapneumonic effusion patients. Patients aged ≥20 years who were consecutively admitted to the emergency department (ED) of Kaohsiung Chang Gung Memorial Hospital (CGMH) were screened for pleural effusion by chest X-ray, chest computerized tomography (CT), or chest sonography. Patients who agreed to join the study and had sufficient leftover pleural fluid for testing were enrolled within 24 h of admission. Kaohsiung CGMH, a 2692-bed acute-care teaching hospital, is the largest medical center in Southern Taiwan and provides both primary and tertiary referral care. The hospital's Institutional Review Committee on Human Research approved the study protocol, and all patients provided their informed consent.

### 2.2. Clinical Assessment and Treatment

Demographic data was collected and underlying disease and clinical presentation information was recorded. Basic laboratory tests including white blood cell counts (WBC) and quantification of inflammatory biomarkers were conducted as part of routine evaluation at the time of ED admission. Pleural fluid was collected within 24 h, and lactic dehydrogenase (LDH), pH level, and leukocyte count were checked daily. The extra pleural fluid was reserved for cell-free DNA measurement, and blood was drawn three more times (Day 1, Day 4, and Day 7) to evaluate change over time in serum cell-free DNA levels. Treatment plans were based on disease severity and agreed upon by the attending physician and each patient. All PPE patients received thoracostomy tube drainage and some patients received further surgical debridement. Personnel conducting the study were blinded to days after admission and disease severity, and the attending physician was blinded to cell-free DNA data. The mortality cases were defined as in-hospital mortality.

### 2.3. Blood and Pleural Fluid Sampling and Assessment of Cell-Free DNA

Blood collection and measurement of serum nuclear and mitochondrial DNA were performed as in our previous study [11]. Pleural fluid was collected in plain tubes and centrifuged for 10 min at 3000 rpm soon after thoracocentesis. Spun fluid was transferred into 1.5 ml clear polypropylene tubes with care taken not to disturb the possible buffy coat layer. The newly separated aliquots were centrifuged for another 10 min at 10,000 rpm. The upper portion of each pleural fluid sample was removed by Pasteur pipette, placed into a fresh clear tube, and stored frozen at -20°C until extraction. DNA was extracted from 200-*μ*l pleural fluid samples using a QIAamp Blood Kit (Qiagen, Frederick, MD) using the “blood and body fluid protocol” based on the manufacturer's instructions. The exact amount of fluid used was documented for calculation of DNA concentrations. The same methodology was used for analysis of blood samples.

Pleural fluid DNA was quantified using real-time quantitative polymerase chain reaction (RT-PCR) (Roche Lightcycler; Roche, Lewes, UK) of the* β-globin* and* MT-ND2* genes based on continuous measurements of SYBR Green fluorescence (SYBR Green dye binds to double-stranded DNA generated during PCR). The* β-globin* gene is present in all nucleated cells of the body, while the* MT-ND2* gene is specific to mitochondrial DNA [14, 15]. The *β*-globin PCR system used the following amplification primers: *β*- globin-354F (5′-GTG CAC CTG ACT CCT GAG GAG A-3′) and *β*-globin- 455R (5′-CCT TGA TAC CAA CCT GCC CAG-3′). The 101-base-pair amplicon was detected using primer sequences and verified in the GenBank database (accession number U01317). The MT-ND2 PCR system used the following amplification primers: MT-ND2-156F (5′-CAC AGA AGC TGC CAT CAA GTA-3′) and MT-ND2-245R (5′-CCG GAG AGT ATA TTG TTG AAG AG-3′). The 90-base-pair amplicon was detected using primer sequences and verified in the GenBank database (accession number NC012920).

All tests were performed in a quality-controlled central laboratory at Chang Gung Memorial Hospital as mentioned in our previous study [11].

### 2.4. Definition of Parapneumonic Pleural Effusion and Nonparapneumonic Pleural Effusion

According to the criteria of the American Thoracic Society, parapneumonic pleural effusion is a type of pleural effusion in pneumonia patient, which is characterized by a positive pleural culture, gram stain, or purulent effusion [16]. According to modified Light's criteria, nonparapneumonic pleural effusion is defined as a transudate with varying etiologies including clinical findings of liver cirrhosis, heart failure, and chronic renal failure [5]. In our study, all patients without the evidence of PPE were defined as non-PPE patients. And the etiology of non-PPE was diagnosed according to the final diagnosis.

### 2.5. Statistical Analyses

Data are expressed as interquartile ranges or percentages. Univariate analyses of continuous variables were performed using the nonparametric Mann-Whitney U test. Categorical variables were compared using the *χ*^2^ test or Fisher's exact test, as appropriate. We explored correlations between different biomarkers in serum and pleural fluid by Spearman correlation. Partial correlation, adjusted for age and gender, was used to test the relationship between days after admission and biomarkers. A p value < 0.05 was considered significant. Receiver operating characteristic (ROC) curves were plotted, and area under the curve (AUC) analysis was used to find the diagnostic accuracy of pleural fluid cell-free DNA. Optimal cut-off values were determined by selecting levels that balanced highest sensitivity against highest specificity. All statistical analyses were conducted using the Statistical Package for the Social Sciences for Windows (software version 20.0; IBM Corp., Armonk, NY, USA).

## 3. Results

### 3.1. Baseline Characteristics of Study Patients

Of the 38 patients enrolled, 22 were diagnosed with PPE and 16 with non-PPE (NPPE). Among the NPPE patients, six were admitted with diagnoses related to liver cirrhosis, six had underlying malignancies, three had congestive heart failure, and one was admitted with unknown etiology. Among six patients with malignancy, three patients had lung cancer, one had pancreatic cancer with lung metastasis, one had esophageal cancer with lung metastasis, and the last one had cholangiocarcinoma. Four of them diagnosed based on pleural cytology report and the other two were clinical diagnosed.  Table 1 shows that the two groups of patients do not have significant differences in age or gender distribution. In the NPPE patient group, there were higher percentages of liver cirrhosis (38% versus 9%) and congestive heart failure (19% versus 0%), though the differences did not reach statistical significance. In clinical presentation, blood pressure, respiratory rate, and heart rates were not different between the two groups, but fever was more prevalent in PPE patients than in NPPE (59% versus 19%, p=0.02). All PPE patients received drainage treatment on the day of thoracocentesis. Of them, four received further surgical intervention during admission. Overall, PPE patients spent more days admitted compared to NPPE patients, but the difference was not statistically significant. In-hospital mortality rates were almost the same in both groups (14% versus 13%).

### 3.2. Serum and Pleural Fluid Biomarkers between PPE and NPPE Patients

**Figure 1:**
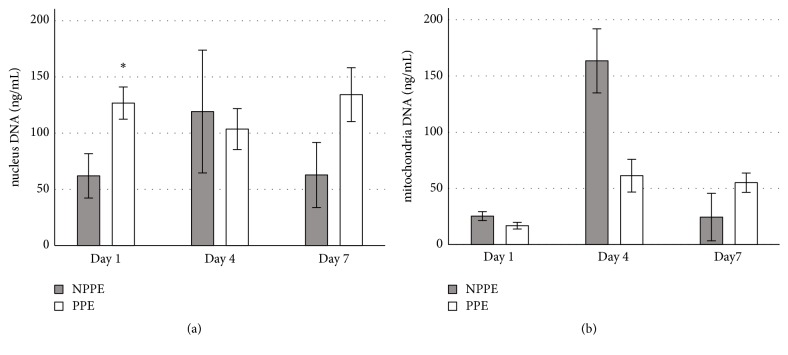
(a). Series serum level of free cell nucleus DNA between parapneumonic pleural effusion (PPE) and nonparapneumonic pleural effusion (NPPE) patients, data represented as mean±SE, and *∗* means *P* < 0.05. (b). Series serum level of free cell mitochondria DNA between parapneumonic pleural effusion (PPE) and nonparapneumonic pleural effusion (NPPE) patients, data represented as mean±SE, and *∗* means *P* < 0.05.

Table 2 shows that, in blood samples, only WBC, C-reactive protein (CRP) and cell-free nuclear DNA were significantly different at Day 1 between PPE and NPPE patients. Figures 1(a) and 1(b) indicate that cell-free nuclear or mitochondrial DNA levels in serum could not distinguish PPE from NPPE during the first 7 days after admission. In pleural fluid, all inflammatory biomarkers including WBC counts, neutrophil percentage, pH, LDH levels, and cell-free DNA were significantly different between PPE and NPPE patients. Spearman correlation tests to evaluate efficacy of these biomarkers, with a focus on cell-free DNA, found that nuclear DNA in pleural fluid correlated strongly with mitochondrial DNA (rho=0.925, P<0.001), WBC count (rho=0.68, P<0.001), neutrophil percentage (rho=0.70, P<0.001), pH (rho= -0.53, P=0.001), LDH level (rho=0.84, P<0.001), and nuclear DNA in serum on Day 1 (rho= 0.60, P<0.001). Mitochondrial DNA in pleural fluid also correlated strongly with WBC count (rho=0.62, P<0.001), neutrophil percentage (rho=0.62, P<0.001), pH (rho= -0.46, P=0.004), LDH level (rho=0.79, P<0.001), and nuclear (rho= 0.65, P<0.001) and mitochondrial (rho= 0.37, P=0.02) DNA in serum on Day 1.

We further evaluate these biomarkers in correlation with number of days after admission in PPE patients (Table 3). With adjustments for age and gender, we found that only pleural fluid nuclear (r= 0.464, P=0.039) and mitochondrial (r=0.538, P=0.014) DNA levels correlated with days of admission.

### 3.3. Serum and Pleural Fluid Cell-Free DNA in Prediction of Parapneumonic Effusion

**Figure 2:**
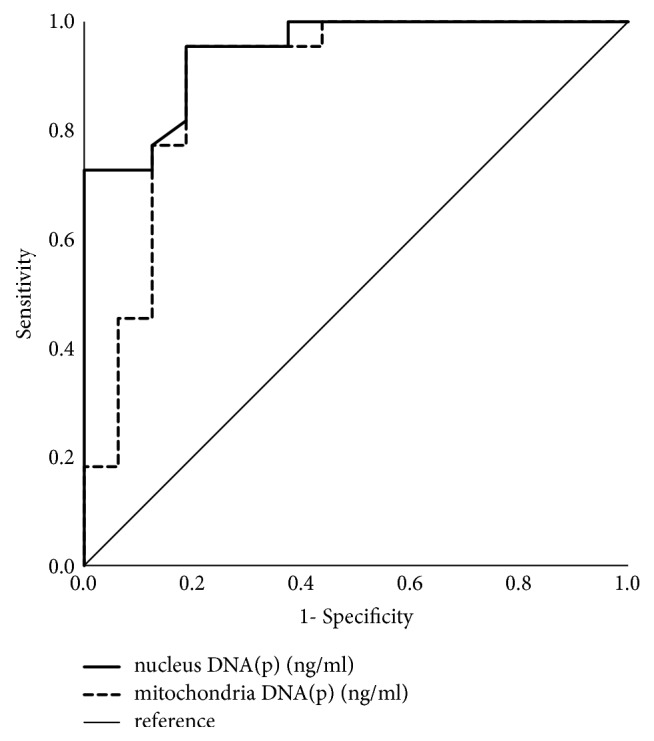
Receiver operating characteristic (ROC) curve of pleural effusion cell-free DNA in diagnosis of parapneumonic pleural effusion.

The efficacy of cell-free DNA in predicting parapneumonic effusion was evaluated by assessing ROC curves. As in Table 4 and Figure 2, pleural fluid nuclear DNA had an AUC of 0.945 (95% confidence interval 0.879-1.000), pleural fluid mitochondrial DNA had an AUC of 0.889 (95% confidence interval 0.769-1.000), and serum nuclear DNA had an AUC of 0.804 (95% confidence interval 0.660-0.948). With optimal cut-off values of 134.9 ng/ml for pleural fluid nuclear DNA and 17.8 ng/ml for mitochondrial DNA, we obtained 96% sensitivity and 81% specificity in the diagnosis of PPE. With a cut-off value of 41.8 ng/ml, serum nuclear DNA exhibited 86% sensitivity and 69% specificity. The level of blood and pleural fluid WBC and neutrophil percentage were also analyzed by ROC curve for comparison. Pleural fluid WBC and neutrophil had AUC of 0.893 (95% confidence interval 0.758-1.000) and 0.845 (95% confidence interval 0.658-1.000), respectively. And blood WBC and neutrophil had AUC of 0.757(95% confidence interval 0.591-0.924) and 0.645 (95% confidence interval 0.461-0.829), respectively. Although they both had good AUC, pleural fluid cell-free DNA had higher AUC and better accuracy in diagnosis of PPE.

## 4. Discussion

To the best of our knowledge, this is the first study to examine both serum and pleural fluid nuclear and mitochondria DNA for diagnosis of PPE that produced the following major findings: First, serum nuclear DNA and pleural fluid nuclear and mitochondrial DNA can be used to diagnose PPE with adequate sensitivity and specificity. Second, pleural fluid nuclear and mitochondrial DNA levels correlated with that of all other biomarkers in pleural fluid, and with their own DNA levels in serum, although pleural fluid nuclear and mitochondrial DNA were the only two biomarkers that correlated with days after admission, a variable that could represent the severity of disease in PPE patients. Third, although serum nuclear DNA at Day 1 could be used to distinguish PPE, it did not correlate with days after admission, and its levels were less than one-tenth of pleural fluid nuclear DNA, which is as different as LDH in clinical use.

Two studies have evaluated pleural fluid cell-free DNA for diagnosis of PPE [10, 13]. While we used the* β-globin* gene to represent nuclear DNA and the* ND2* gene to represent mitochondria DNA, both previous studies used only the* β-globin* gene to represent cell-free DNA. They also lacked data on cell-free DNA in serum for comparison. In the study by Chan et al., 50 patients were enrolled, but only 11 belonged to the infected pleural effusion group and 9 of those 11 were diagnosed with pulmonary tuberculosis, leaving only two patients with PPE [10]. Chan et al. concluded that pleural fluid cell-free DNA had the potential to differentiate transudate from exudate with an AUC of 0.95. This is quite different from our study, which primarily focused on PPE. Santotoribio et al. enrolled 78 pleural effusion patients, but only 16 had PPE. There were also significantly more patients with malignant pleural effusion (30/62) in his non-PPE group, which produced a wide range of pleural fluid cell-free DNA levels. The AUC was 0.904 for diagnosis of PPE and 0.994 for differentiation of transudate and exudate. These results are compatible with our AUC of 0.945 for diagnosis of PPE, as we had more non-PPE patients with transudate.

Benlloch et al. demonstrated the prognostic utility of cell-free DNA in pleural fluid and serum of cancer patients with pleural effusion [17]. This study used the gene coding for the RNA subunit of human ribonuclease P as a target for detecting cell-free DNA and found a correlation (r=0.3, P=0.05) between cell-free DNA levels in pleural fluid and serum, as did our study. In this study, the median pleural fluid cell-free DNA level of 70 cancer patients was lower than their median serum DNA level. This result is compatible with our data in malignant pleural effusion patients, unlike the study by Santotoribio et al., in which cell-free DNA was extremely high in pleural fluid.

Serum cell-free DNA has demonstrated applications in various aspects of different diseases [18], but there are still no studies that evaluate serum cell-free DNA for diagnosis of PPE. In our previous study of severe sepsis patients, we found that a serum nuclear DNA level of 1012 ng/ml exhibited 82% sensitivity and 82% specificity and a serum mitochondrial DNA level of 198 ng/ml exhibited 91% sensitivity and 72% specificity in predicting mortality [11]. In these patients, cell-free DNA gradually decreased over the first 7 days. This differs from our PPE study, which predicted PPE at a relatively low level of serum nuclear DNA (41.8 ng/ml), and which showed no consistent change in levels over the first 7 days after admission.

In plasma, the majority of cell-free DNA comes from leukocytes. Cell-free DNA is rapidly degraded by nucleases present in the blood and is eliminated by the liver and kidneys. The half-life of cell-free DNA varies from 4 min to several h depending on physical conditions [19]. In our study, most PPE patients did not meet the criteria for severe sepsis, which means they experienced more localized inflammatory reactions. In the pleural space and lung parenchyma, destroyed cells and apoptotic leukocytes would release large amounts of cell-free DNA, which would accumulate in the pleural space. This would dramatically increase pleural fluid cell-free DNA, as shown in our study. Whether pleural fluid cell-free DNA would have access to the circulation to be eliminated from the blood needs further investigation. However, Benlloch et al. and our study both demonstrate that serum cell-free DNA correlated with pleural fluid cell-free DNA [17]. This gives us a hint of communication between pleural fluid and blood. It could also merely indicate a correlation in severity of inflammatory reactions at two different sites.

According to the American College of Chest Physicians' guidelines for treatment of PPE, PPE could be categorized into uncomplicated PPE, complicated PPE, and empyema due to shared location (pleural space), bacteriology, and pH criteria [2]. In our study, all PPE patients initially receive drainage therapy, which means they presented with at least a moderate amount of effusion. Four of them underwent surgical debridement later due to poor drainage, lobulated effusion, or empyema change. From that perspective, we enrolled a higher percentage of complicated PPE patients in this study. As the treatment plan was dependent on response to antibiotics and drainage, we used days after admission to represent severity of PPE in our study. If we could predict upon admission which patients would need longer stays with standard treatment regimens, we might better identify patients who would benefit from more, aggressive interventions early on. Our study showed that pleural fluid nuclear and mitochondrial DNA may be a good indicator of these patients.

Some studies used inflammatory biomarkers like CRP and procalcitonin (PCT) in serum or pleural fluid to predict PPE [8, 20, 21]. In a study of PCT, serum levels had better predictive values than pleural fluid levels, but all AUC values were around 0.84-0.75, which is not very impressive. In a meta-analysis study, Zou et al. found that serum and pleural fluid PCT had low sensitivity and specificity in diagnosis of PPE, and only serum CRP had high rule-in value [22]. Compared to our data, pleural fluid cell-free DNA could be used to diagnose PPE with better sensitivity and specificity than its serum level or other biomarkers. In addition, both CRP and PCT had lower cut-off values in pleural fluid than in serum for diagnosis of PPE. This is different from our study, as cell-free DNA came from the destroyed and apoptotic cells or leukocytes expected to accumulate in the pleural space. This factor made pleural fluid cell-free DNA more suitable for diagnosis of PPE than the other biomarkers. Whether it is suitable to distinguish PPE from other etiologies of pleural effusion with abundant cells inside needs further investigation.

Although we demonstrated that pleural fluid nuclear and mitochondrial DNA and serum nuclear DNA could predict PPE with high sensitivity and specificity, our study faced several limitations. First, compared to our cohort of 22 patients with PPE, we included fewer non-PPE patients, and we found it difficult to perform subgroup analyses with different etiologies of pleural effusion. Second, we did not check the other target inflammatory biomarkers in pleural fluid simultaneously to obtain head-to-head comparisons with cell-free DNA. Finally, variations in AUC or in sensitivity and specificity between studies could stem from different selection methods used to recruit the various groups of patients.

In conclusion, our study revealed that pleural fluid nuclear and mitochondrial DNA were useful for diagnosis of PPE and for gauging severity of the disease. Serum nuclear DNA from Day 1 serum could also distinguish non-PPE patients, but with less accuracy than pleural fluid, and serum levels did not correlate with severity.

## Figures and Tables

**Table 1:** Baseline characteristics of parapneumonic and non-parapneumonic pleural effusion patients.

	Non-parapneumonic effusion	Parapneumonic effusion	*P* value
	n=16	n=22	
Gender(male)(n (%))	12(75%)	15(68%)	0.73
Age (years old)	61(51, 75)	57(51, 63)	0.29
Major comorbidity			
Congestive heart failure (n (%))	3(19%)	0(0%)	0.07
Diabetes mellitus (n (%))	2(13%)	9(41%)	0.08
Hypertension (n (%))	5(31%)	8(36%)	1.00
Cerebrovascular disease (n (%))	0(0%)	3(14%)	0.23
Cancer (n (%))	7(44%)	5(23%)	0.29
Liver cirrhosis (n (%))	6(38%)	2(9%)	0.05
COPD (n (%))	1(6%)	2(9%)	1.00
Clinical presentation			
Fever episode (n (%))	3(19%)	13(59%)	0.02^*∗*^
Systolic blood pressure (mmHg)	173(113, 197)	153(129, 188)	0.67
Diastolic blood pressure(mmHg)	94(82, 111)	88(78, 102)	0.28
Pulse rate (times/minute)	96(74, 123)	115(103, 127)	0.10
Respiratory rate (times/minute)	19(18, 22)	20(18, 20)	0.95
Days of admission(days)	14(13, 17)	20(15, 31)	0.10
In hospital mortality (n (%))	2(13%)	3(14%)	1.00

COPD, chronic obstructive pulmonary disease.

Data was expressed as median (interquartile range Q1, Q3).

^*∗*^ *P* < 0.05.

**Table 2:** Serum and pleural effusion laboratory data of parapneumonic and nonparapneumonic pleural effusion patients.

	Non-parapneumonic effusion	Parapneumonic effusion	*P* value
	n=16	n=22	
Serum level			
White blood cell count (×10^9^/L)	9.8(4.9, 14.2)	15.7(11.5, 19.4)	0.007^*∗*^
Neutrophil (%)	79.5(62.2, 82.2)	81.2(72.9, 86.1)	0.13
C reactive protein (mg/L)	75.0(12.7, 125.5)	177.6(129.9, 264.6)	<0.001^*∗*^
Procalcitonin (ng/mL)	0.18(0.09, 1.09)	0.92(0.27, 3.94)	0.20
LDH (U/L)	227.0(208.0, 248.0)	221.0(180.8, 271.0)	0.54
nucleus DNA_day1_ (ng/mL)	38.6(23.2, 73.7)	125.5(60.0, 281.3)	0.002^*∗*^
mitochondria DNA_day1_ (ng/mL)	21.2(15.0, 36.8)	31.8(14.5, 126.0)	0.322
Pleural fluid			
White blood cell count (×10^6^/L)	0.37(0.14, 0.95)	2.90(1.35, 11.01)	<0.001^*∗*^
Neutrophil (%)	8.0(3.5, 23.8)	84.0(60.0, 89.0)	<0.001^*∗*^
pH level	8.0(7.9, 8.3)	7.0(6.8, 8.1)	0.02^*∗*^
LDH (U/L)	92.5(72.5, 415.5)	1035.5(593.0, 2645.8)	<0.001^*∗*^
nucleus DNA (ng/mL)	1.8 (0.2, 59.1)	10300.0(1420.0, 14200.0)	<0.001^*∗*^
mitochondria DNA (ng/mL)	1.8(1.1, 13.7)	243.0(95.7, 491.5)	<0.001^*∗*^

LDH: lactic dehydrogenase; DNA: deoxyribonucleic acid.

Data was expressed as median (interquartile range Q1, Q3).

^*∗*^ *P* < 0.05.

**Table 3:** The partial correlation between days of admission and inflammatory biomarkers in serum and pleural effusion in parapneumonic effusion patients.

	WBC(p)	pH(p)	LDH(p)	Nucleus DNA(p)	Mitochondria DNA(p)	CRP(s)	WBC(s)	Nucleus DNA_day1_ (s)
*r*	-0.121	-0.393	0.358	0.464	0.538	-0.257	-0.203	-0.06
*P* value	0.613	0.086	0.121	0.039^*∗*^	0.014^*∗*^	0.274	0.390	0.81

*r* = Pearson correlation coefficient; WBC: white blood cell count; LDH: lactic dehydrogenase; DNA: deoxyribonucleic acid; CRP: C reactive protein, (s): serum; (p): pleural fluid; ^*∗*^*P* < 0.05.

**Table 4:** Receiver operating characteristic curve analysis of pleural effusion cell free DNA in diagnosis of parapneumonic effusion and its sensitivity and specificity with suggested cut-off value.

	AUC	*P* value	95% confidence interval		Cut off value	Sensitivity	Specificity
			Lower limit	Upper limit			
Nucleus DNA(p) (ng/ml)	0.945	<0.001^*∗*^	0.879	1.000	134.9	0.96	0.81
Mitochondrial DNA(p) (ng/ml)	0.889	<0.001^*∗*^	0.769	1.000	17.8	0.96	0.81
Nucleus DNA(s) (ng/ml)	0.804	0.002^*∗*^	0.660	0.948	41.8	0.86	0.69
White blood cell count (p) (×10^6^/L)	0.893	0.002^*∗*^	0.758	1.000	1.285	0.81	0.90
Neutrophil(p) (%)	0.845	0.007^*∗*^	0.658	1.000	58	0.80	0.90
White blood cell count (s) (×10^9^/L)	0.757	0.007^*∗*^	0.591	0.924	11.5	0.77	0.75
Neutrophil(s) (%)	0.645	0.132	0.461	0.829	81	0.64	0.69

AUC: area under curve; DNA: deoxyribonucleic acid; (s): serum; (p): pleural fluid; ^*∗*^*P* < 0.05.

## Data Availability

The data used to support the findings of this study are available from the corresponding author upon request.
